# (*E*)-(4-Chloro­benzyl­idene){[(1*R*,4a*S*,10a*R*)-7-isopropyl-1,4a-dimethyl-1,2,3,4,4a,9,10,10a-octa­hydro-1-phenanthryl]­methyl}amine

**DOI:** 10.1107/S1600536809013245

**Published:** 2009-04-25

**Authors:** Yu-Xiang Chen, Zhen-Dong Zhao, Yu-Min Wang, Liang-Wu Bi

**Affiliations:** a Institute of Chemical Industry of Forest Products, Chinese Academy of Forestry, Nanjing 210042, People’s Republic of China

## Abstract

The title compound, C_27_H_34_ClN, has been synthesized from 4-chloro­benzaldehyde and dehydro­abietylamine. There are two unique mol­ecules in the unit cell. Each mol­ecule has three chiral centres, which exhibit *R*, *S* and *R* absolute configurations. The two cyclo­hexane rings form a *trans* ring junction with classical chair and half-chair conformations.

## Related literature

For the background to dehydro­abietylamine, an important chiral diterpenic amine with a hydro­phanthrene structure, see: Gottstein & Cheney (1965 ▶). For the biological activity of dehydro­abietylamine derivatives, see: Wilkerson *et al.* (1993 ▶).
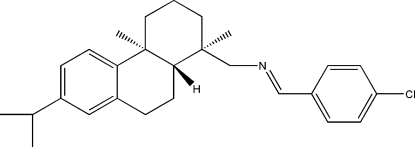


## Experimental

### 

#### Crystal data

C_27_H_34_ClN
*M* *_r_* = 408.00
Triclinic, 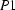

*a* = 5.9251 (13) Å
*b* = 10.783 (2) Å
*c* = 19.163 (4) Å
α = 77.402 (4)°
β = 85.281 (4)°
γ = 78.224 (4)°
*V* = 1168.8 (4) Å^3^
*Z* = 2
Mo *K*α radiation
μ = 0.18 mm^−1^
*T* = 273 K
0.15 × 0.12 × 0.08 mm

#### Data collection

Bruker APEX CCD area-detector diffractometer
Absorption correction: multi-scan (*SADABS*; Sheldrick, 1996 ▶) *T*_min_ = 0.974, *T*_max_ = 0.986
6211 measured reflections
4891 independent reflections
3236 reflections with *I* > 2σ(*I*)
*R*_int_ = 0.024

#### Refinement

*R*[*F* ^2^ > 2σ(*F* ^2^)] = 0.062
*wR*(*F* ^2^) = 0.188
*S* = 1.02
4891 reflections
518 parameters
1347 restraints
H-atom parameters constrained
Δρ_max_ = 0.31 e Å^−3^
Δρ_min_ = −0.31 e Å^−3^
Absolute structure: Flack (1983 ▶) 794 Friedel pairs
Flack parameter: 0.19 (12)

### 

Data collection: *SMART* (Bruker, 1997 ▶); cell refinement: *SAINT* (Bruker, 1997 ▶); data reduction: *SAINT*; program(s) used to solve structure: *SHELXS97* (Sheldrick, 2008 ▶); program(s) used to refine structure: *SHELXL97* (Sheldrick, 2008 ▶); molecular graphics: *SHELXTL* (Sheldrick, 2008 ▶); software used to prepare material for publication: *SHELXTL*.

## Supplementary Material

Crystal structure: contains datablocks I, global. DOI: 10.1107/S1600536809013245/at2759sup1.cif

Structure factors: contains datablocks I. DOI: 10.1107/S1600536809013245/at2759Isup2.hkl

Additional supplementary materials:  crystallographic information; 3D view; checkCIF report

## Figures and Tables

**Fig. 1.**
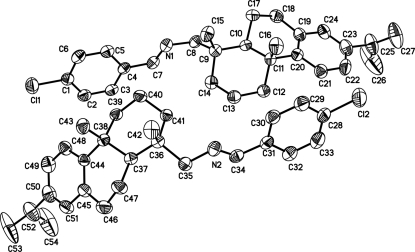
A view of the two unique molecules of (I), showing displacement ellipsoids at the 30% probability level and the atom numbering scheme.

## supplementary crystallographic information

### Comment

Dehydroabietylamine is an important chiral diterpenic amine with the hydrophanthrene structure (Gottstein *et al.*, 1965). Dehydroabietylamine derivatives exhibit a wide range of biological activity (Wilkerson *et al.*, 1993). Although much attention has been paid to the bioactivity of dehydroabietylamine derivatives, the crystal structure of the title compound has not yet been reported and we describe its structure here, Fig 1.  The compound crystallises with two unique molecules in the triclinic unit cell. Each molecule contains four rings. The two cyclohexane rings with a classical chair and half-chair conformations form a *trans* ring junction. The two methyl groups attached to the cyclohexane rings are in axial positions. The carbon atoms C11 and C18 in the cyclohexane ring and the atoms in the conjoint benzene ring are in the same plane.

### Experimental

A mixture of 4 - chlorobenzaldehyde (0.03 mol), dehydroabietylamine (0.03 mol) and ethanol (150 ml) was refluxed for 4 h. The resulting mixture was cooled to room temperature, then filtered. The precipitate was washed with water and ethanol. Upon recrystallization from ethanol, colorless crystals of the title compound were obtained.

### Refinement

All H atoms bonded to the C atoms were placed geometrically at the distances of 0.93–0.98 Å and included in the refinement in the riding approximation with *U*_iso_(H) = 1.2 or 1.5*U*_eq_ of the carrier atom.

### Crystal data

**Table:**

C_27_H_34_ClN	*Z* = 2
*M**_r_* = 408.00	*F*(000) = 440
Triclinic, *P*1	*D*_x_ = 1.159 Mg m^−^^3^
Hall symbol: P 1	Mo *K*α radiation, λ = 0.71073 Å
*a* = 5.9251 (13) Å	Cell parameters from 1191 reflections
*b* = 10.783 (2) Å	θ = 2.4–18.6°
*c* = 19.163 (4) Å	µ = 0.18 mm^−^^1^
α = 77.402 (4)°	*T* = 273 K
β = 85.281 (4)°	Block, colourless
γ = 78.224 (4)°	0.15 × 0.12 × 0.08 mm
*V* = 1168.8 (4) Å^3^	

### Data collection

**Table:**

Bruker APEX CCD area-detector diffractometer	4891 independent reflections
Radiation source: fine-focus sealed tube	3236 reflections with *I* > 2σ(*I*)
graphite	*R*_int_ = 0.024
φ and ω scans	θ_max_ = 25.1°, θ_min_ = 2.0°
Absorption correction: multi-scan (*SADABS*; Sheldrick, 1996)	*h* = −6→7
*T*_min_ = 0.974, *T*_max_ = 0.986	*k* = −10→12
6211 measured reflections	*l* = −20→22

### Refinement

**Table:**

Refinement on *F*^2^	Hydrogen site location: inferred from neighbouring sites
Least-squares matrix: full	H-atom parameters constrained
*R*[*F*^2^ > 2σ(*F*^2^)] = 0.062	*w* = 1/[σ^2^(*F*_o_^2^) + (0.1124*P*)^2^] where *P* = (*F*_o_^2^ + 2*F*_c_^2^)/3
*wR*(*F*^2^) = 0.188	(Δ/σ)_max_ < 0.001
*S* = 1.02	Δρ_max_ = 0.31 e Å^−^^3^
4891 reflections	Δρ_min_ = −0.31 e Å^−^^3^
518 parameters	Extinction correction: *SHELXL*, Fc^*^=kFc[1+0.001xFc^2^λ^3^/sin(2θ)]^-1/4^
1347 restraints	Extinction coefficient: 0.012 (4)
Primary atom site location: structure-invariant direct methods	Absolute structure: Flack (1983) 794 Friedel pairs
Secondary atom site location: difference Fourier map	Flack parameter: 0.19 (12)

### Special details

**Table:**

Geometry. All esds (except the esd in the dihedral angle between two l.s. planes) are estimated using the full covariance matrix. The cell esds are taken into account individually in the estimation of esds in distances, angles and torsion angles; correlations between esds in cell parameters are only used when they are defined by crystal symmetry. An approximate (isotropic) treatment of cell esds is used for estimating esds involving l.s. planes.
Refinement. Refinement of F^2^ against ALL reflections. The weighted R-factor wR and goodness of fit S are based on F^2^, conventional R-factors R are based on F, with F set to zero for negative F^2^. The threshold expression of F^2^ > 2sigma(F^2^) is used only for calculating R-factors(gt) etc. and is not relevant to the choice of reflections for refinement. R-factors based on F^2^ are statistically about twice as large as those based on F, and R- factors based on ALL data will be even larger.

### Fractional atomic coordinates and isotropic or equivalent isotropic displacement parameters (Å^2^)

**Table:**

	*x*	*y*	*z*	*U*_iso_*/*U*_eq_	
Cl1	0.3555 (3)	0.97493 (17)	0.04545 (10)	0.0952 (6)	
Cl2	0.7197 (5)	−0.2151 (2)	0.68356 (11)	0.1210 (9)	
N1	0.2137 (8)	0.6837 (4)	0.3893 (3)	0.0618 (12)	
N2	0.8562 (8)	0.0563 (4)	0.3353 (3)	0.0641 (13)	
C1	0.2792 (10)	0.8976 (6)	0.1296 (3)	0.0635 (14)	
C2	0.0903 (11)	0.8392 (6)	0.1394 (3)	0.0670 (14)	
H2	0.0042	0.8414	0.1005	0.080*	
C3	0.0289 (10)	0.7777 (5)	0.2063 (3)	0.0615 (13)	
H3	−0.0997	0.7388	0.2123	0.074*	
C4	0.1535 (9)	0.7718 (5)	0.2660 (3)	0.0564 (12)	
C5	0.3454 (10)	0.8302 (5)	0.2544 (3)	0.0613 (13)	
H5	0.4365	0.8252	0.2925	0.074*	
C6	0.4031 (11)	0.8957 (6)	0.1868 (4)	0.0690 (14)	
H6	0.5268	0.9386	0.1805	0.083*	
C7	0.0872 (10)	0.7066 (5)	0.3372 (3)	0.0578 (12)	
H7	−0.0550	0.6809	0.3441	0.069*	
C8	0.1358 (9)	0.6147 (5)	0.4584 (3)	0.0587 (13)	
H8A	−0.0224	0.6056	0.4552	0.070*	
H8B	0.1375	0.6660	0.4941	0.070*	
C9	0.2843 (9)	0.4798 (5)	0.4834 (3)	0.0548 (12)	
C10	0.1857 (9)	0.4181 (5)	0.5573 (3)	0.0504 (11)	
H10	0.0220	0.4239	0.5497	0.061*	
C11	0.2793 (9)	0.2695 (5)	0.5851 (3)	0.0534 (11)	
C12	0.2433 (10)	0.2005 (6)	0.5256 (3)	0.0614 (12)	
H12A	0.3102	0.1092	0.5395	0.074*	
H12B	0.0791	0.2079	0.5208	0.074*	
C13	0.3507 (10)	0.2556 (6)	0.4531 (3)	0.0638 (13)	
H13A	0.5168	0.2415	0.4565	0.077*	
H13B	0.3182	0.2105	0.4176	0.077*	
C14	0.2572 (10)	0.3985 (5)	0.4296 (3)	0.0584 (12)	
H14A	0.0946	0.4106	0.4207	0.070*	
H14B	0.3352	0.4304	0.3848	0.070*	
C15	0.5355 (9)	0.4965 (6)	0.4838 (3)	0.0677 (15)	
H15A	0.5721	0.5540	0.4405	0.102*	
H15B	0.6366	0.4138	0.4870	0.102*	
H15C	0.5547	0.5320	0.5242	0.102*	
C16	0.5323 (10)	0.2337 (7)	0.6044 (4)	0.0767 (17)	
H16A	0.5671	0.1446	0.6285	0.115*	
H16B	0.5608	0.2875	0.6353	0.115*	
H16C	0.6282	0.2466	0.5616	0.115*	
C17	0.1851 (11)	0.4910 (6)	0.6172 (3)	0.0666 (13)	
H17A	0.1479	0.5836	0.5988	0.080*	
H17B	0.3358	0.4701	0.6378	0.080*	
C18	0.0017 (11)	0.4489 (6)	0.6743 (3)	0.0716 (14)	
H18A	0.0182	0.4805	0.7169	0.086*	
H18B	−0.1503	0.4889	0.6565	0.086*	
C19	0.0174 (10)	0.3059 (6)	0.6941 (3)	0.0651 (13)	
C20	0.1373 (9)	0.2239 (6)	0.6528 (3)	0.0591 (12)	
C21	0.1391 (11)	0.0909 (6)	0.6766 (3)	0.0714 (14)	
H21	0.2206	0.0325	0.6500	0.086*	
C22	0.0201 (12)	0.0457 (7)	0.7397 (4)	0.0825 (16)	
H22	0.0287	−0.0430	0.7556	0.099*	
C23	−0.1107 (12)	0.1300 (8)	0.7793 (4)	0.0816 (15)	
C24	−0.1036 (12)	0.2553 (7)	0.7569 (3)	0.0808 (15)	
H24	−0.1833	0.3128	0.7843	0.097*	
C25	−0.2448 (14)	0.0768 (9)	0.8472 (4)	0.103 (2)	
H25	−0.2794	0.1562	0.8663	0.124*	
C26	−0.4642 (19)	0.0709 (14)	0.8369 (6)	0.159 (4)	
H26A	−0.4804	−0.0180	0.8457	0.239*	
H26B	−0.5021	0.1117	0.7884	0.239*	
H26C	−0.5664	0.1148	0.8691	0.239*	
C27	−0.1150 (18)	0.0008 (11)	0.9025 (4)	0.138	
H27A	−0.0715	−0.0859	0.8946	0.207*	
H27B	−0.2038	0.0004	0.9467	0.207*	
H27C	0.0212	0.0349	0.9048	0.207*	
C28	0.7244 (12)	−0.1573 (6)	0.5922 (4)	0.0716 (15)	
C29	0.8802 (12)	−0.0856 (6)	0.5604 (4)	0.0777 (15)	
H29	0.9899	−0.0697	0.5876	0.093*	
C30	0.8785 (11)	−0.0359 (6)	0.4880 (3)	0.0708 (15)	
H30	0.9863	0.0139	0.4668	0.085*	
C31	0.7175 (11)	−0.0593 (6)	0.4465 (3)	0.0634 (13)	
C32	0.5608 (12)	−0.1338 (6)	0.4801 (4)	0.0719 (14)	
H32	0.4513	−0.1510	0.4533	0.086*	
C33	0.5641 (13)	−0.1832 (6)	0.5528 (4)	0.0799 (16)	
H33	0.4582	−0.2337	0.5749	0.096*	
C34	0.7038 (11)	−0.0028 (5)	0.3698 (3)	0.0625 (12)	
H34	0.5793	−0.0104	0.3456	0.075*	
C35	0.8203 (10)	0.1093 (5)	0.2595 (3)	0.0603 (13)	
H35A	0.6616	0.1113	0.2496	0.072*	
H35B	0.9192	0.0530	0.2317	0.072*	
C36	0.8725 (9)	0.2479 (5)	0.2359 (3)	0.0526 (11)	
C37	0.7780 (9)	0.3039 (5)	0.1601 (3)	0.0538 (11)	
H37	0.6178	0.2914	0.1652	0.065*	
C38	0.7591 (9)	0.4506 (5)	0.1303 (3)	0.0527 (11)	
C39	0.6276 (10)	0.5216 (5)	0.1872 (3)	0.0548 (12)	
H39A	0.6286	0.6134	0.1719	0.066*	
H39B	0.4682	0.5108	0.1906	0.066*	
C40	0.7289 (10)	0.4734 (5)	0.2607 (3)	0.0585 (13)	
H40A	0.8839	0.4909	0.2584	0.070*	
H40B	0.6365	0.5204	0.2943	0.070*	
C41	0.7370 (9)	0.3309 (5)	0.2873 (3)	0.0522 (11)	
H41A	0.5805	0.3151	0.2942	0.063*	
H41B	0.8078	0.3041	0.3335	0.063*	
C42	1.1337 (9)	0.2383 (6)	0.2398 (3)	0.0732 (17)	
H42A	1.2125	0.2028	0.2007	0.110*	
H42B	1.1859	0.1832	0.2843	0.110*	
H42C	1.1659	0.3231	0.2370	0.110*	
C43	0.9946 (10)	0.4930 (7)	0.1118 (3)	0.0677 (15)	
H43A	0.9708	0.5807	0.0852	0.102*	
H43B	1.0892	0.4374	0.0835	0.102*	
H43C	1.0700	0.4873	0.1551	0.102*	
C44	0.6259 (9)	0.4867 (5)	0.0612 (3)	0.0558 (11)	
C45	0.6104 (11)	0.3978 (6)	0.0217 (3)	0.0655 (12)	
C46	0.7238 (13)	0.2559 (7)	0.0427 (3)	0.0797 (15)	
H46A	0.6046	0.2047	0.0572	0.096*	
H46B	0.8074	0.2287	0.0013	0.096*	
C47	0.8876 (12)	0.2288 (6)	0.1026 (3)	0.0698 (14)	
H47A	1.0318	0.2552	0.0844	0.084*	
H47B	0.9200	0.1368	0.1232	0.084*	
C48	0.5188 (11)	0.6147 (6)	0.0351 (3)	0.0697 (13)	
H48	0.5286	0.6772	0.0608	0.084*	
C49	0.3999 (12)	0.6518 (8)	−0.0268 (3)	0.0820 (16)	
H49	0.3353	0.7386	−0.0430	0.098*	
C50	0.3752 (12)	0.5605 (8)	−0.0655 (4)	0.0812 (15)	
C51	0.4841 (11)	0.4363 (7)	−0.0415 (3)	0.0777 (15)	
H51	0.4752	0.3743	−0.0676	0.093*	
C52	0.2384 (14)	0.6007 (9)	−0.1327 (4)	0.0992 (19)	
H52	0.1981	0.6947	−0.1369	0.119*	
C53	0.3623 (18)	0.5923 (12)	−0.1967 (4)	0.146 (3)	
H53A	0.2625	0.6299	−0.2359	0.219*	
H53B	0.4229	0.5031	−0.1977	0.219*	
H53C	0.4872	0.6382	−0.2008	0.219*	
C54	0.0157 (17)	0.5672 (13)	−0.1229 (5)	0.143 (3)	
H54A	0.0318	0.4748	−0.1098	0.215*	
H54B	−0.0647	0.5988	−0.1667	0.215*	
H54C	−0.0699	0.6055	−0.0856	0.215*	

### Atomic displacement parameters (Å^2^)

**Table:**

	*U*^11^	*U*^22^	*U*^33^	*U*^12^	*U*^13^	*U*^23^
Cl1	0.1210 (15)	0.0791 (12)	0.0781 (11)	−0.0183 (11)	0.0226 (10)	−0.0107 (9)
Cl2	0.186 (2)	0.0886 (14)	0.0764 (12)	−0.0194 (15)	0.0093 (13)	−0.0028 (11)
N1	0.057 (3)	0.054 (3)	0.073 (3)	−0.010 (2)	0.001 (3)	−0.012 (2)
N2	0.065 (3)	0.049 (3)	0.072 (3)	0.000 (2)	−0.005 (3)	−0.006 (2)
C1	0.067 (3)	0.051 (3)	0.071 (3)	−0.005 (2)	0.004 (3)	−0.015 (2)
C2	0.067 (3)	0.056 (3)	0.077 (3)	−0.007 (2)	−0.011 (3)	−0.016 (3)
C3	0.061 (3)	0.050 (3)	0.075 (3)	−0.010 (2)	−0.008 (2)	−0.016 (2)
C4	0.055 (3)	0.042 (2)	0.072 (3)	−0.006 (2)	−0.003 (2)	−0.016 (2)
C5	0.056 (3)	0.053 (3)	0.074 (3)	−0.005 (2)	−0.009 (2)	−0.014 (2)
C6	0.062 (3)	0.059 (3)	0.086 (3)	−0.015 (2)	−0.002 (3)	−0.014 (3)
C7	0.048 (2)	0.049 (2)	0.074 (3)	−0.005 (2)	−0.006 (2)	−0.011 (2)
C8	0.054 (3)	0.049 (3)	0.071 (3)	−0.007 (2)	−0.001 (2)	−0.012 (2)
C9	0.041 (2)	0.057 (2)	0.065 (3)	−0.005 (2)	−0.002 (2)	−0.013 (2)
C10	0.045 (2)	0.050 (2)	0.059 (2)	−0.0099 (19)	−0.002 (2)	−0.016 (2)
C11	0.045 (2)	0.055 (2)	0.057 (2)	−0.0049 (19)	−0.002 (2)	−0.009 (2)
C12	0.059 (3)	0.058 (3)	0.065 (3)	−0.004 (2)	0.000 (2)	−0.016 (2)
C13	0.062 (3)	0.058 (3)	0.068 (3)	−0.001 (2)	0.002 (2)	−0.018 (2)
C14	0.050 (2)	0.058 (3)	0.063 (3)	−0.003 (2)	0.007 (2)	−0.015 (2)
C15	0.045 (3)	0.073 (3)	0.084 (3)	−0.017 (3)	−0.005 (3)	−0.007 (3)
C16	0.056 (3)	0.079 (4)	0.084 (4)	−0.003 (3)	−0.008 (3)	−0.001 (3)
C17	0.071 (3)	0.064 (3)	0.068 (3)	−0.012 (2)	−0.006 (2)	−0.019 (2)
C18	0.074 (3)	0.075 (3)	0.064 (3)	−0.008 (2)	0.005 (2)	−0.020 (2)
C19	0.062 (3)	0.069 (3)	0.062 (2)	−0.010 (2)	−0.001 (2)	−0.013 (2)
C20	0.054 (2)	0.061 (2)	0.061 (2)	−0.011 (2)	−0.006 (2)	−0.009 (2)
C21	0.069 (3)	0.073 (3)	0.068 (3)	−0.006 (2)	−0.005 (2)	−0.010 (2)
C22	0.079 (3)	0.084 (3)	0.076 (3)	−0.019 (3)	0.000 (3)	0.004 (3)
C23	0.070 (3)	0.096 (3)	0.068 (3)	−0.009 (3)	0.003 (3)	−0.003 (3)
C24	0.075 (3)	0.094 (3)	0.067 (3)	−0.011 (3)	0.002 (3)	−0.012 (3)
C25	0.079 (4)	0.128 (4)	0.086 (4)	−0.014 (4)	0.003 (3)	0.006 (4)
C26	0.122 (6)	0.211 (8)	0.125 (6)	−0.046 (6)	0.007 (5)	0.017 (6)
C27	0.124	0.165	0.086	−0.002	0.026	0.019
C28	0.084 (3)	0.049 (3)	0.078 (3)	−0.008 (3)	−0.001 (3)	−0.011 (3)
C29	0.086 (3)	0.066 (3)	0.083 (3)	−0.017 (3)	−0.010 (3)	−0.014 (3)
C30	0.075 (3)	0.061 (3)	0.078 (3)	−0.020 (3)	−0.004 (3)	−0.011 (3)
C31	0.068 (3)	0.051 (2)	0.072 (3)	−0.015 (2)	0.001 (2)	−0.015 (2)
C32	0.075 (3)	0.060 (3)	0.082 (3)	−0.019 (3)	−0.001 (3)	−0.013 (3)
C33	0.085 (3)	0.062 (3)	0.088 (3)	−0.013 (3)	0.011 (3)	−0.011 (3)
C34	0.063 (2)	0.051 (2)	0.074 (3)	−0.007 (2)	−0.003 (2)	−0.016 (2)
C35	0.060 (3)	0.053 (3)	0.064 (3)	0.000 (2)	−0.005 (2)	−0.012 (2)
C36	0.046 (2)	0.051 (2)	0.060 (2)	−0.005 (2)	−0.001 (2)	−0.015 (2)
C37	0.050 (2)	0.055 (2)	0.058 (2)	−0.0077 (19)	0.000 (2)	−0.019 (2)
C38	0.048 (2)	0.056 (2)	0.056 (2)	−0.012 (2)	0.003 (2)	−0.015 (2)
C39	0.055 (2)	0.051 (2)	0.059 (2)	−0.007 (2)	0.002 (2)	−0.019 (2)
C40	0.059 (3)	0.060 (3)	0.061 (3)	−0.009 (2)	0.003 (2)	−0.024 (2)
C41	0.051 (2)	0.052 (2)	0.056 (2)	−0.014 (2)	0.000 (2)	−0.014 (2)
C42	0.045 (3)	0.082 (4)	0.082 (4)	−0.001 (3)	−0.001 (3)	−0.005 (3)
C43	0.057 (3)	0.078 (3)	0.065 (3)	−0.018 (3)	0.001 (3)	−0.006 (3)
C44	0.052 (2)	0.061 (2)	0.055 (2)	−0.013 (2)	−0.001 (2)	−0.013 (2)
C45	0.068 (3)	0.069 (3)	0.058 (2)	−0.010 (2)	−0.002 (2)	−0.016 (2)
C46	0.092 (3)	0.077 (3)	0.069 (3)	−0.005 (3)	−0.007 (3)	−0.021 (2)
C47	0.075 (3)	0.062 (3)	0.068 (3)	0.005 (2)	−0.002 (2)	−0.022 (2)
C48	0.068 (3)	0.074 (3)	0.063 (2)	−0.006 (2)	−0.002 (2)	−0.013 (2)
C49	0.074 (3)	0.089 (3)	0.070 (3)	0.002 (3)	−0.004 (3)	−0.005 (3)
C50	0.070 (3)	0.102 (3)	0.065 (3)	−0.009 (3)	−0.009 (3)	−0.009 (3)
C51	0.080 (3)	0.095 (3)	0.061 (3)	−0.021 (3)	−0.007 (3)	−0.018 (3)
C52	0.089 (4)	0.128 (4)	0.073 (3)	−0.007 (3)	−0.011 (3)	−0.015 (3)
C53	0.131 (6)	0.207 (8)	0.081 (5)	−0.020 (6)	−0.009 (5)	0.003 (6)
C54	0.108 (6)	0.211 (8)	0.098 (5)	−0.015 (6)	−0.018 (5)	−0.013 (6)

### Geometric parameters (Å, °)

**Table:**

Cl1—C1	1.717 (6)	C27—H27A	0.9600
Cl2—C28	1.726 (7)	C27—H27B	0.9600
N1—C7	1.250 (7)	C27—H27C	0.9600
N1—C8	1.460 (7)	C28—C29	1.347 (9)
N2—C34	1.269 (7)	C28—C33	1.366 (10)
N2—C35	1.455 (7)	C29—C30	1.373 (9)
C1—C6	1.364 (8)	C29—H29	0.9300
C1—C2	1.373 (8)	C30—C31	1.385 (9)
C2—C3	1.367 (8)	C30—H30	0.9300
C2—H2	0.9300	C31—C32	1.380 (8)
C3—C4	1.394 (8)	C31—C34	1.465 (8)
C3—H3	0.9300	C32—C33	1.380 (9)
C4—C5	1.389 (8)	C32—H32	0.9300
C4—C7	1.454 (7)	C33—H33	0.9300
C5—C6	1.385 (8)	C34—H34	0.9300
C5—H5	0.9300	C35—C36	1.551 (8)
C6—H6	0.9300	C35—H35A	0.9700
C7—H7	0.9300	C35—H35B	0.9700
C8—C9	1.538 (8)	C36—C42	1.537 (8)
C8—H8A	0.9700	C36—C41	1.543 (8)
C8—H8B	0.9700	C36—C37	1.549 (7)
C9—C14	1.528 (8)	C37—C47	1.531 (8)
C9—C15	1.536 (7)	C37—C38	1.545 (7)
C9—C10	1.550 (7)	C37—H37	0.9800
C10—C17	1.526 (8)	C38—C44	1.532 (7)
C10—C11	1.572 (7)	C38—C39	1.536 (7)
C10—H10	0.9800	C38—C43	1.546 (7)
C11—C16	1.525 (8)	C39—C40	1.520 (7)
C11—C20	1.532 (8)	C39—H39A	0.9700
C11—C12	1.541 (8)	C39—H39B	0.9700
C12—C13	1.528 (8)	C40—C41	1.502 (8)
C12—H12A	0.9700	C40—H40A	0.9700
C12—H12B	0.9700	C40—H40B	0.9700
C13—C14	1.508 (8)	C41—H41A	0.9700
C13—H13A	0.9700	C41—H41B	0.9700
C13—H13B	0.9700	C42—H42A	0.9600
C14—H14A	0.9700	C42—H42B	0.9600
C14—H14B	0.9700	C42—H42C	0.9600
C15—H15A	0.9600	C43—H43A	0.9600
C15—H15B	0.9600	C43—H43B	0.9600
C15—H15C	0.9600	C43—H43C	0.9600
C16—H16A	0.9600	C44—C45	1.366 (9)
C16—H16B	0.9600	C44—C48	1.396 (8)
C16—H16C	0.9600	C45—C51	1.416 (8)
C17—C18	1.540 (9)	C45—C46	1.520 (9)
C17—H17A	0.9700	C46—C47	1.505 (9)
C17—H17B	0.9700	C46—H46A	0.9700
C18—C19	1.490 (9)	C46—H46B	0.9700
C18—H18A	0.9700	C47—H47A	0.9700
C18—H18B	0.9700	C47—H47B	0.9700
C19—C20	1.368 (9)	C48—C49	1.371 (9)
C19—C24	1.411 (8)	C48—H48	0.9300
C20—C21	1.405 (8)	C49—C50	1.391 (10)
C21—C22	1.392 (8)	C49—H49	0.9300
C21—H21	0.9300	C50—C51	1.360 (10)
C22—C23	1.385 (10)	C50—C52	1.512 (10)
C22—H22	0.9300	C51—H51	0.9300
C23—C24	1.334 (10)	C52—C53	1.388 (11)
C23—C25	1.532 (10)	C52—C54	1.425 (12)
C24—H24	0.9300	C52—H52	0.9800
C25—C26	1.347 (12)	C53—H53A	0.9600
C25—C27	1.372 (11)	C53—H53B	0.9600
C25—H25	0.9800	C53—H53C	0.9600
C26—H26A	0.9600	C54—H54A	0.9600
C26—H26B	0.9600	C54—H54B	0.9600
C26—H26C	0.9600	C54—H54C	0.9600
			
C7—N1—C8	118.9 (5)	H27B—C27—H27C	109.5
C34—N2—C35	116.7 (5)	C29—C28—C33	120.5 (6)
C6—C1—C2	119.9 (6)	C29—C28—Cl2	120.6 (6)
C6—C1—Cl1	120.2 (5)	C33—C28—Cl2	118.8 (5)
C2—C1—Cl1	119.8 (5)	C28—C29—C30	120.5 (7)
C3—C2—C1	119.9 (6)	C28—C29—H29	119.7
C3—C2—H2	120.0	C30—C29—H29	119.7
C1—C2—H2	120.0	C29—C30—C31	120.6 (6)
C2—C3—C4	121.9 (5)	C29—C30—H30	119.7
C2—C3—H3	119.1	C31—C30—H30	119.7
C4—C3—H3	119.1	C32—C31—C30	117.9 (6)
C5—C4—C3	117.0 (5)	C32—C31—C34	120.0 (6)
C5—C4—C7	121.4 (5)	C30—C31—C34	122.0 (5)
C3—C4—C7	121.7 (5)	C33—C32—C31	121.0 (7)
C6—C5—C4	121.0 (6)	C33—C32—H32	119.5
C6—C5—H5	119.5	C31—C32—H32	119.5
C4—C5—H5	119.5	C28—C33—C32	119.4 (6)
C1—C6—C5	120.2 (6)	C28—C33—H33	120.3
C1—C6—H6	119.9	C32—C33—H33	120.3
C5—C6—H6	119.9	N2—C34—C31	122.2 (6)
N1—C7—C4	122.6 (5)	N2—C34—H34	118.9
N1—C7—H7	118.7	C31—C34—H34	118.9
C4—C7—H7	118.7	N2—C35—C36	112.4 (5)
N1—C8—C9	113.4 (4)	N2—C35—H35A	109.1
N1—C8—H8A	108.9	C36—C35—H35A	109.1
C9—C8—H8A	108.9	N2—C35—H35B	109.1
N1—C8—H8B	108.9	C36—C35—H35B	109.1
C9—C8—H8B	108.9	H35A—C35—H35B	107.9
H8A—C8—H8B	107.7	C42—C36—C41	110.9 (5)
C14—C9—C15	111.1 (5)	C42—C36—C37	114.4 (4)
C14—C9—C8	107.0 (4)	C41—C36—C37	108.2 (4)
C15—C9—C8	107.7 (5)	C42—C36—C35	108.0 (5)
C14—C9—C10	108.4 (4)	C41—C36—C35	108.1 (4)
C15—C9—C10	114.2 (4)	C37—C36—C35	107.1 (4)
C8—C9—C10	108.2 (4)	C47—C37—C38	109.7 (4)
C17—C10—C9	115.9 (4)	C47—C37—C36	115.0 (4)
C17—C10—C11	109.9 (4)	C38—C37—C36	117.8 (4)
C9—C10—C11	116.3 (4)	C47—C37—H37	104.2
C17—C10—H10	104.4	C38—C37—H37	104.2
C9—C10—H10	104.4	C36—C37—H37	104.2
C11—C10—H10	104.4	C44—C38—C39	110.6 (4)
C16—C11—C20	107.2 (4)	C44—C38—C37	108.2 (4)
C16—C11—C12	109.3 (5)	C39—C38—C37	107.5 (4)
C20—C11—C12	110.2 (4)	C44—C38—C43	107.4 (4)
C16—C11—C10	115.2 (5)	C39—C38—C43	109.2 (5)
C20—C11—C10	108.0 (4)	C37—C38—C43	113.9 (5)
C12—C11—C10	106.9 (4)	C40—C39—C38	113.3 (4)
C13—C12—C11	113.1 (5)	C40—C39—H39A	108.9
C13—C12—H12A	109.0	C38—C39—H39A	108.9
C11—C12—H12A	109.0	C40—C39—H39B	108.9
C13—C12—H12B	109.0	C38—C39—H39B	108.9
C11—C12—H12B	109.0	H39A—C39—H39B	107.7
H12A—C12—H12B	107.8	C41—C40—C39	111.9 (5)
C14—C13—C12	111.3 (5)	C41—C40—H40A	109.2
C14—C13—H13A	109.4	C39—C40—H40A	109.2
C12—C13—H13A	109.4	C41—C40—H40B	109.2
C14—C13—H13B	109.4	C39—C40—H40B	109.2
C12—C13—H13B	109.4	H40A—C40—H40B	107.9
H13A—C13—H13B	108.0	C40—C41—C36	113.1 (4)
C13—C14—C9	114.5 (5)	C40—C41—H41A	109.0
C13—C14—H14A	108.6	C36—C41—H41A	108.9
C9—C14—H14A	108.6	C40—C41—H41B	108.9
C13—C14—H14B	108.6	C36—C41—H41B	109.0
C9—C14—H14B	108.6	H41A—C41—H41B	107.8
H14A—C14—H14B	107.6	C36—C42—H42A	109.5
C9—C15—H15A	109.5	C36—C42—H42B	109.5
C9—C15—H15B	109.5	H42A—C42—H42B	109.5
H15A—C15—H15B	109.5	C36—C42—H42C	109.5
C9—C15—H15C	109.5	H42A—C42—H42C	109.5
H15A—C15—H15C	109.5	H42B—C42—H42C	109.5
H15B—C15—H15C	109.5	C38—C43—H43A	109.5
C11—C16—H16A	109.5	C38—C43—H43B	109.5
C11—C16—H16B	109.5	H43A—C43—H43B	109.5
H16A—C16—H16B	109.5	C38—C43—H43C	109.5
C11—C16—H16C	109.5	H43A—C43—H43C	109.5
H16A—C16—H16C	109.5	H43B—C43—H43C	109.5
H16B—C16—H16C	109.5	C45—C44—C48	117.1 (5)
C10—C17—C18	107.3 (5)	C45—C44—C38	122.4 (5)
C10—C17—H17A	110.3	C48—C44—C38	120.5 (5)
C18—C17—H17A	110.3	C44—C45—C51	120.0 (6)
C10—C17—H17B	110.3	C44—C45—C46	122.7 (5)
C18—C17—H17B	110.3	C51—C45—C46	117.3 (6)
H17A—C17—H17B	108.5	C47—C46—C45	113.3 (6)
C19—C18—C17	113.5 (5)	C47—C46—H46A	108.9
C19—C18—H18A	108.9	C45—C46—H46A	108.9
C17—C18—H18A	108.9	C47—C46—H46B	108.9
C19—C18—H18B	108.9	C45—C46—H46B	108.9
C17—C18—H18B	108.9	H46A—C46—H46B	107.7
H18A—C18—H18B	107.7	C46—C47—C37	109.1 (5)
C20—C19—C24	119.5 (6)	C46—C47—H47A	109.9
C20—C19—C18	122.6 (5)	C37—C47—H47A	109.9
C24—C19—C18	117.9 (6)	C46—C47—H47B	109.9
C19—C20—C21	117.6 (5)	C37—C47—H47B	109.9
C19—C20—C11	123.1 (5)	H47A—C47—H47B	108.3
C21—C20—C11	119.2 (5)	C49—C48—C44	122.6 (7)
C22—C21—C20	120.7 (6)	C49—C48—H48	118.7
C22—C21—H21	119.7	C44—C48—H48	118.7
C20—C21—H21	119.7	C48—C49—C50	120.4 (7)
C23—C22—C21	121.4 (7)	C48—C49—H49	119.8
C23—C22—H22	119.3	C50—C49—H49	119.8
C21—C22—H22	119.3	C51—C50—C49	117.3 (6)
C24—C23—C22	116.8 (6)	C51—C50—C52	122.2 (7)
C24—C23—C25	123.3 (8)	C49—C50—C52	120.5 (7)
C22—C23—C25	119.8 (7)	C50—C51—C45	122.4 (7)
C23—C24—C19	124.0 (7)	C50—C51—H51	118.8
C23—C24—H24	118.0	C45—C51—H51	118.8
C19—C24—H24	118.0	C53—C52—C54	119.0 (9)
C26—C25—C27	124.8 (8)	C53—C52—C50	116.6 (7)
C26—C25—C23	114.4 (7)	C54—C52—C50	113.6 (7)
C27—C25—C23	116.2 (7)	C53—C52—H52	101.1
C26—C25—H25	97.1	C54—C52—H52	101.1
C27—C25—H25	97.1	C50—C52—H52	101.1
C23—C25—H25	97.1	C52—C53—H53A	109.5
C25—C26—H26A	109.5	C52—C53—H53B	109.5
C25—C26—H26B	109.5	H53A—C53—H53B	109.5
H26A—C26—H26B	109.5	C52—C53—H53C	109.5
C25—C26—H26C	109.5	H53A—C53—H53C	109.5
H26A—C26—H26C	109.5	H53B—C53—H53C	109.5
H26B—C26—H26C	109.5	C52—C54—H54A	109.5
C25—C27—H27A	109.5	C52—C54—H54B	109.5
C25—C27—H27B	109.5	H54A—C54—H54B	109.5
H27A—C27—H27B	109.5	C52—C54—H54C	109.5
C25—C27—H27C	109.5	H54A—C54—H54C	109.5
H27A—C27—H27C	109.5	H54B—C54—H54C	109.5
